# Bending Flexibility of Moso Bamboo (*Phyllostachys Edulis*) with Functionally Graded Structure

**DOI:** 10.3390/ma12122007

**Published:** 2019-06-23

**Authors:** Xin Wei, Haiying Zhou, Fuming Chen, Ge Wang

**Affiliations:** 1 International Centre for Bamboo and Rattan, Beijing 100102, China; weixin199522@163.com (X.W.); 15288423522@163.com (H.Z.); 2 National Forestry and Grassland Administration/Beijing Co-build Key Laboratory of Bamboo and Rattan Science & Technology, Beijing 100102, China

**Keywords:** bending flexibility, graded structure, directional structure, vascular bundles, parenchyma cells

## Abstract

As one of the most renewable and sustainable resources on Earth, bamboo with its high flexibility has been used in the fabrication of a wide variety of composite structures due to its properties. A bamboo-based winding composite (BWC) is an innovative bamboo product which has revolutionized pipe structures and their applications throughout China as well as improving their impact on the environment. However, as a natural functionally graded composite, the flexibility mechanism of bamboo has not yet been fully understood. Here, the bending stiffness method based on the cantilever beam principle was used to investigate the gradient and directional bending flexibility of bamboo (*Phyllostachys edulis*) slivers under different loading Types during elastic stages. Results showed that the graded distribution and gradient variation of cell size of the fibers embedded in the parenchyma cells along the thickness of the bamboo culm was mainly responsible for the exhibited gradient bending flexibility of bamboo slivers, whereas the shape and size difference of the vascular bundles from inner to outer layers played a critical role in directional bending flexibility. A validated rule of mixture was used to fit the bending stiffness under different loading Types as a function of fiber volume fraction. This work provides insights to the bionic preparation and optimization of high-performance BWC pipes.

## 1. Introduction

Bamboo refers to a large variety of grass species that are widely distributed in tropical and subtropical regions throughout the world. It is also one of the fastest growing and highest growing herbaceous plants in the world [1]. Usually, it takes only three years for many bamboo species to grow from seedlings to more than ten meters as an adult plant [2]. This growth period is extremely short compared to most woods. However, bamboo contains 40–48% cellulose, 22–27% hemicellulose, and 25–30% lignin, which is similar to the ratios reported to be in both softwood and hardwood, which allows bamboo exhibits similar physical properties [3]. These rapidly renewable, low-cost, high-strength, and high-rigidity characteristics make bamboo desirable material that is widely used in a variety of fields, including construction materials, pulp and paper, biomass energy, etc. These properties of bamboo demonstrate the potential possibilities of it being a substitute for wood under the policy of prohibiting the felling of natural forests in China [4].

**Figure 1:**
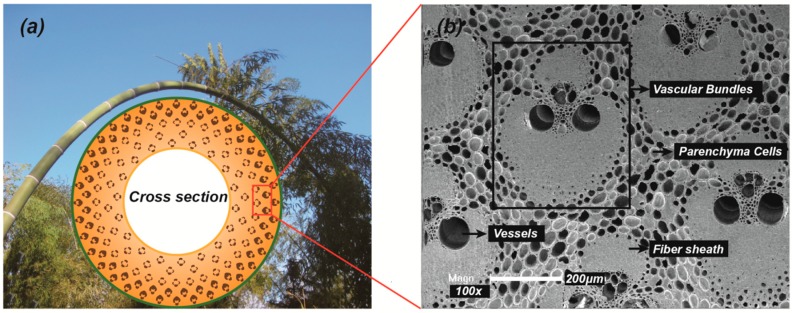
(**a**) Excellent bending flexibility of bamboo with hollow structure and FG structure in cross section under heavy snow; (**b**) Field emission scanning electron microscope (FESEM) micrograph of microstructure of bamboo culm with different constituents.

**Figure 2:**
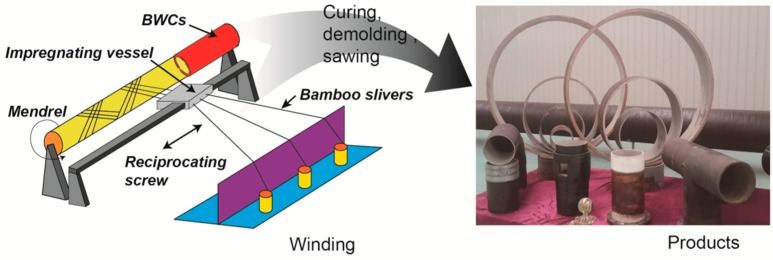
Processing and products of BWC pipes winding with bamboo slivers.

In order to adapt to harsh natural conditions such as wind and snow during natural growth (Figure 1a), bamboo has formed a superior hollow multi-node structure in the growth direction and functionally graded (FG) structure on the wall layer in the long-term biological evolution process (Figure 1b), which endows it with a unique bending flexibility feature that "bends without damage and rebounds quickly". Taking full advantage of the excellent bending flexibility, with the processing technology of weaving, molding and winding in carbon or glass fiber composites, the bamboo-based heterotypic engineered components have been developed and applied using bamboo sliver as the raw material, especially for bamboo-based winding composites (BWC) pipes (Figure 2). These BWC pipes have become an important starting point for the development of the forestry industry in China, where they can fulfill the roles as drainage pipelines, the body of high-speed railway cars, modern buildings, and so on [5].

The excellent bending flexibility characteristics of bamboo are the result of the combination of bamboo’s own properties, environmental factors, and dimensional factors. Compared with wood of the same moisture content and size, bamboo is prone to more flexible deformation and large curvature winding, which is caused by the difference in microstructure between bamboo and wood. Bamboo is a typical two-phase composite material (Figure 1b): The enhanced phase fibers in vascular bundles are the source of bamboo strength and stiffness; the matrix-phase parenchyma cells absorb energy through plastic deformation to increase the toughness and ductility of the structure; the hierarchical interfaces between the two Types of phase cells and the cell wall layers allows bamboo to transmit stress well [6,7]. Therefore, the gradient structures of vascular bundles embedded in parenchyma cells and perfect combination (hierarchical interface) of two phase cell structures the main reason for the excellent flexibility of bamboo [8]. However, Yu research group [9] found that longitudinal tensile modulus of bamboo was lower than the value evaluated by rule of mixture and showed that the interface between the two phases was weaker. Therefore, the mechanism of synergistic deformation of vascular bundles and parenchyma cells under stress was still unclear. It is still a problem in the field of basic bamboo properties research that the test method which is an evaluation index of the internal mechanism of bending flexibility characteristics of bamboo under the gradient distribution of vascular bundles on the wall of bamboo culm cannot resolve.

The bending flexibility of the materials or engineered components (the reciprocal of the bending stiffness EI) refers to the deflection caused by the unit force within the range of elastic deformation. The current test method of flexibility includes 2-point [10], 3-point [11] and 4-point bending test [12] for obtaining the bending modulus E of the material, whereas the section moment of inertia I is obtained by the test size. In order to simulate the loading conditions of bamboo in natural environmental conditions, such as lateral wind, the bending stiffness method based on the cantilever beam principle can be applied to the study of the bending flexibility of thin bamboo slivers for BWC pipes with a thickness of 1 mm. At present, this method has been used for slender and thin materials in the millimeter or micrometer scale, such as single plant single fiber [13], polymer film [14], and paper and paperboard [15], which is a fast and east test that provides high precision measurements.

Among the efforts focused on the bending properties of bamboo, it appears that differences in strength, modulus, fracture toughness, and failure mode of bamboo pertaining to the gradient distribution of vascular bundle of bamboo have been studied the most extensively [8,16,17,18] whereas the importance of flexibility deformation behavior, alongside their corresponding internal mechanisms interacted with the tissue structure of bamboo, has seen little study due to it being underrated. Therefore, this study aims to clarify the influence and intrinsic mechanism of the directional structure and gradient structure of bamboo vascular bundles on the bending flexibility and introduce a numerical model to predict the gradient flexural behavior along the thicknesses of bamboo culm. This will provide insights to bamboo’s flexible deformation during the natural growth process, bamboo-sliver traditional handicrafts, processing of novel bamboo-based heterotypic engineering component, and bio inspired design of advanced structural and functional materials. Due to this insight and understanding the natural mechanical design principles of bamboo, which can provide theoretical guidance for directional bionic preparation and optimization of high-performance BWC pipes can be known.

## 2. Materials and Methods

### 2.1. Sample Preparation

**Figure 3:**
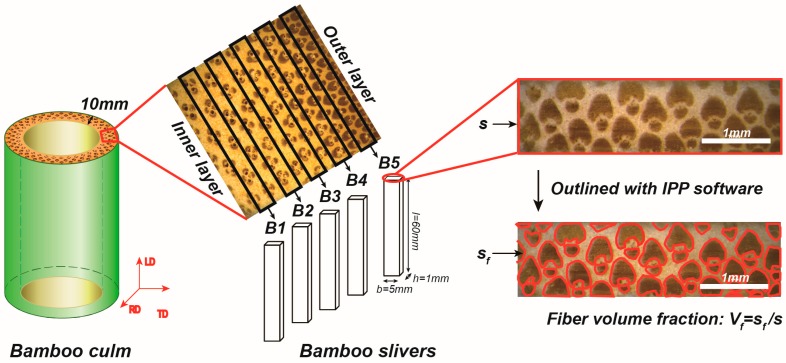
Schematic diagram of processing of samples and calculation of fiber volume fraction (*V_f_*).

Mature (about 4 years old) Moso bamboo (*Phyllostachys edulis)* was collected from bamboo plantations located in Fujian Province China and were selected as the raw materials for this study. Straight and non-defective middle bamboo culm (about 5 m above the ground) was chosen to ensure stable mechanical performance. According to sample size requirements for BWC pipes [5], bamboo culm sections (H = 10 mm) were divided into smooth slivers (5 layers, marking as B1 to B5) with same size (60 × 5 × 1 mm^3^) but different fiber volume fractions from the inner to outer parts (Figure 3). 15 samples were selected for each layer for later tests. All samples were kept at room temperature of 20 °C and relative humidity of 65% until a constant weight (moisture content of 10%), and were pressed under an iron plate to prevent deformation.

### 2.2. Fiber Volume Fraction Calculation

Assuming that the vascular bundles embedded in the parenchyma cells are straight and complete, a clear cross-sectional image of the sample was taken using an optical microscope. A clear sample of the fiber profile in Figure 3 was selected and used to calculate the pixel area using Image Pro Plus 6.0 graphics processing software (Media Cybernetics, Silver spring, MD, USA), marking as *s* and *s_f_*. The fiber volume fraction (*V_f_*) of each sliver was calculated through Equation (1)

(1) $$V_{f}=\frac{s_{f}}{s}$$

### 2.3. Bending Flexibility Test Methods

**Figure 4:**
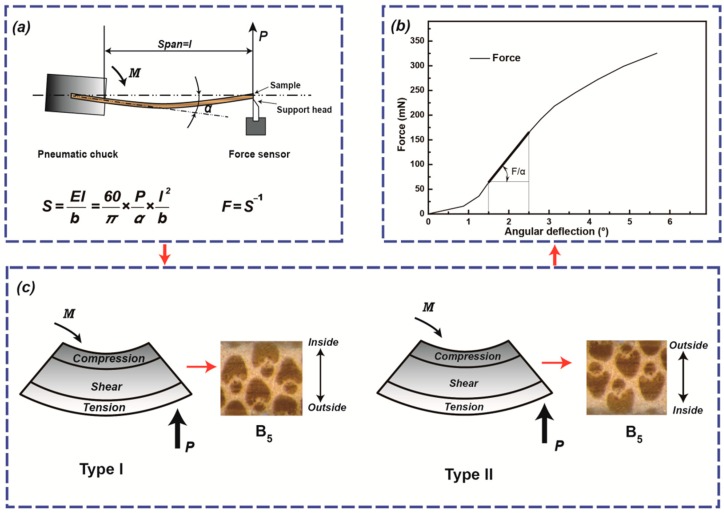
(**a**) the schematic of the bending flexibility test based on the beam-bending principle; (**b**) a typical curve of force versus angular deflection (*P-α*) of specimen with the span thickness ratio (*l/h*) = 50; (**c**) specimen (B5 as an example) loaded by Types I and II.

According to DIN 53121, the bending stiffness method based on the principle of cantilever beam was used to evaluate the bending flexibility of the specimens and was performed on a bending stiffness tester (Lanbo Testing Instrument Co. Ltd., Shenzhen, China). A slower bending speed of 10 °/s and load cell of 10N (Figure 4a) was used for the stiffness test. The maximum span thickness ratio (*l/h* = 50) was selected to minimize the impact of inter-laminar shear strain. The beam-shaped sample was clamped at one end in a clamp that rotated and was subjected to a force, *P*, acting perpendicular to the surface of the sample at the start of the test, at a bending length, *l*, from the clamp. In Figure 4b, a typical curve of force versus angular deflection (*P-α*) of the specimens was recorded. The slope (*P/α*) of the initial linear portion was used to calculate the bending stiffness (*S*) and bending flexibility (*F*) according to Equations (2) and (3).

(2) $$S=\frac{EI}{b}=\frac{60}{\pi }\times \frac{P}{\alpha }\times \frac{l^{2}}{b}$$

(3) $$F=S^{-1}$$

The specimens underwent two Types of loading, which was determined according to the orientation of the arrow shape of the fibers. The arrow shape of the fibers points in the direction of the inside of the bamboo culm the slivers were made from. Type I loading, the fibers arrow shape is pointing upwards so when it is loaded the inside part of the fibers are in compression and the outside part is in tension outside (Figure 4c). Type II loading, is the inverse of Type one loading and reverses the orientation of the fibers by 180 degrees (Figure 4c). 15 specimens were cut from each layer.

## 3. Results and Discussion

### 3.1. Gradient Bending Flexibility

**Figure 5:**
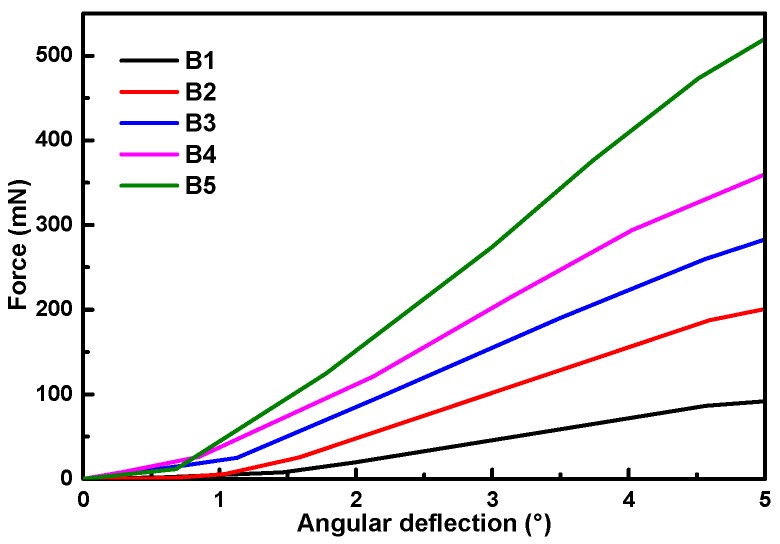
Bending force versus angular deflection (*P-α*) of different layers of bamboo culm.

Figure 1a shows that bamboo is a typical FG structural material where the fibers in the bamboo culm are densely distributed in the outer layers and sparsely scattered in the inner layers. This change in fiber concentration causes a gradient bending behavior of bamboo slivers from inner layer (B1) to outer layer (B5), which is schematically depicted in Figure 5 and summarized in Table 1. In addition, the increase in bending force as the bending angle increases is also shown in Figure 5.

**Figure 6:**
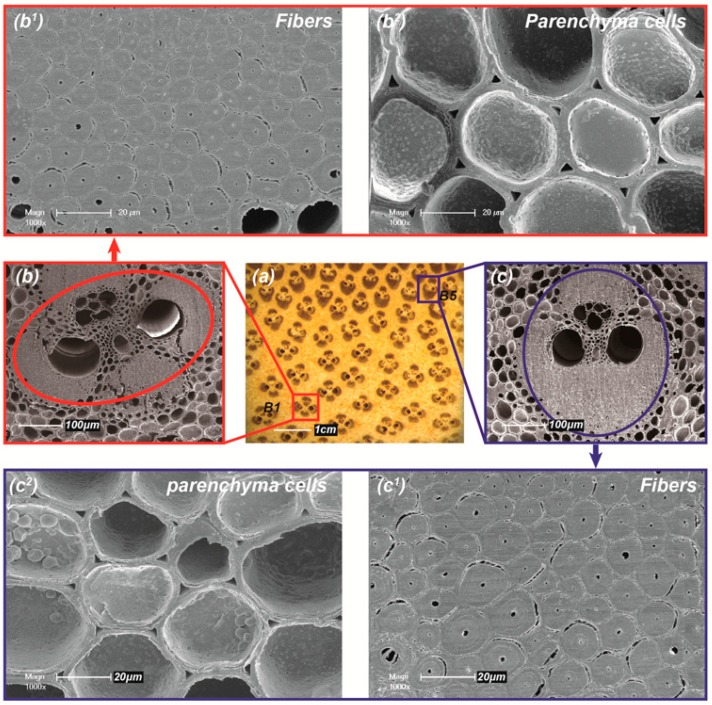
(**a**) Optical microscope picture of FG structure on bamboo culm cross section; the shape of vascular bundles with symmetrical (**b**) and directional (**c**) structure and morphology of fibers (**b^1^**,**c^1^**) and parenchyma cells (**b^2^**,**c^2^**) in the inner layer and outer layer, respectively.

Type I loading (Figure 4c) of bamboo in a natural environment was used to analyze its gradient flexible behavior. The elastic bending behavior characterization of Type I loading demonstrated qualitatively that bamboo slivers (B5) prepared from the outer layer with their high fiber volume fractions (~38.9 ± 6.0%) exhibited high bending stiffness and low bending flexibility, whereas bamboo slivers (B1) prepared from the inner layer, with low fiber volume fractions (~15.5 ± 1.7%), exhibited high bending flexibility and low bending stiffness (Table 1). Further, at the cellular level, the gradient variation along the diameter of cell size is also an important factor for the gradient mechanical behavior of bamboo. As displayed in Figure 6b^1^,b^2^,c^1^,c^2^, the diameter of fibers in the inner layer was slightly larger than that in the outer layer, while the parenchyma cells in the outer layer were larger. 

Since bamboo is a typical FG material (Figure 6a), its hollow cylindrical sclerenchyma fibers in vascular bundles (aggregates of fibers and parenchyma cells) help to contribute to the flexural modulus and bending stiffness of the bamboo slivers, while the matrix phase parenchyma cells with large cavity and thin wall easily absorb energy due to elastic deformation which increases the structural flexibility. The multi-scale weak interfaces between the two-phase cells and the cell wall layers increase flexibility due to interfacial slip. Therefore, the gradient bending flexibility of bamboo slivers can be attributed mainly to gradient distribution and gradient variation of cell size of reinforcing fibers embedded in parenchyma cells along the thickness direction from inner to outer layers. 

The flexible slivers prepared from the bamboos inner layer is used to wind to the inner layers of the bamboo pipe, while the rigid bamboo outer layer is used to wind the outer layer of the pipe. This combination of soft inner and rigid outer layers helps to prepare WPCs and optimize the processing of the pipe structure and its structural bionics purposes. 

### 3.2. Directional Bending Flexibility

**Figure 7:**
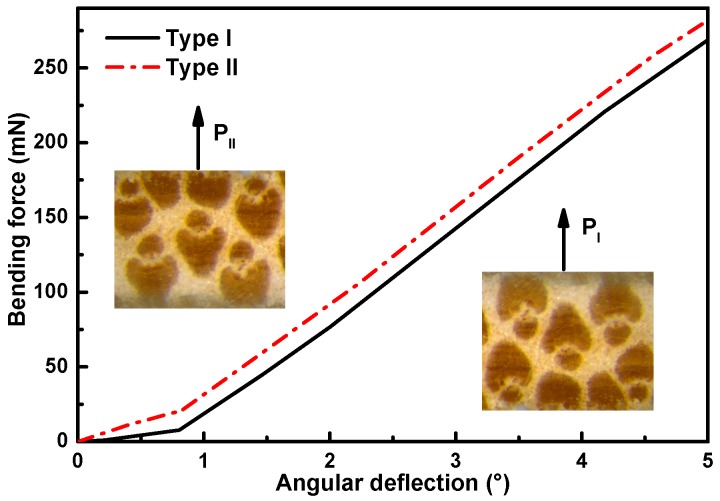
The (*P-α*) curves of B5 layer under loading Type I and II.

**Figure 8:**
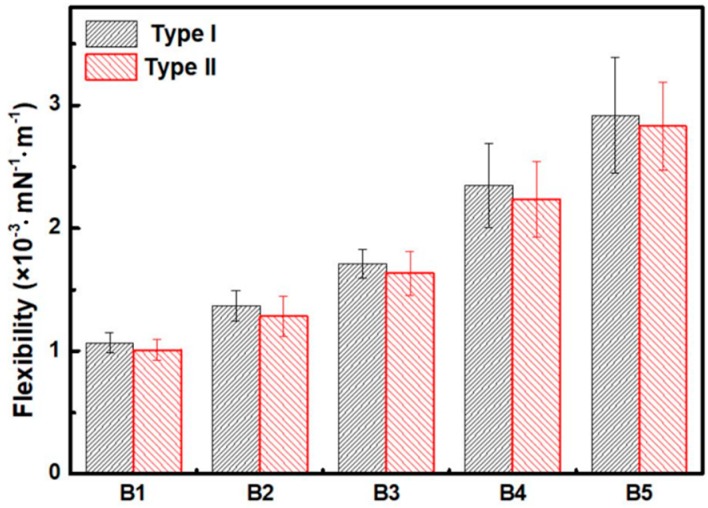
Flexibility of the same sample under different loading Types.

In addition to the gradient bending flexibility due to the FG distribution of fibers along the thickness, the bending flexibility of bamboo slivers in different layers also exhibited obvious directionality, which is schematically displayed in Figure 7 and Figure 8 and listed in Table 1. 

Compared to the Type I, the Type II had a higher slope of the curve. Bamboo slivers from B1 to B5, exhibited slightly lower bending flexibility than under Type I loading, which demonstrated that bamboo had better flexibility to adapt to a natural environment loading Type. Further, B4 and B5 prepared from the outer layers possessed a larger difference of bending flexibility, 6.19% and 5.31%, respectively, whereas B1 and B2 prepared from the inner layers had a lower bending flexibility difference of 3.05% and 4.89%. 

In the cross section of the bamboo culm (Figure 6a), the anatomical shape and size of vascular bundles shows obvious directionality. From inner layer to outer layer, the long axis of the approximately elliptical vascular bundles gradually becomes shorter, and the short axis is shortened, which leads to a semi-open arrow structure (Figure 6c) containing two fibers sheaths (aggregate of fibers) simplified from an open structure (Figure 6b) consisting of four similarly sized and substantially symmetrical fibers sheaths [19]. The smaller difference of flexibility in inner layers (e.g., B1 and B2) in different loading Types was due to the almost elimination of the gradient characteristics in the thickness direction (due to the samples being very thin) and substantially symmetrical structure of the vascular bundles in the inner layers. the higher difference of flexibility in outer layers (e.g., B4 and B5) was due to the bottom of the sample being in tension there were fewer fibers sheaths present under Type I loading (Figure 4a) when compared with the Type II. This meant the directional structure of the vascular bundles lead to the difference of flexibility due to the reduced concentration of vascular bundles present between the two Types of loading.

### 3.3. Modeling and Predicting of the Gradient Bending Flexibility 

In light of the exhibited graded bending behavior along the thickness of the bamboo strips, the classical model given by Equations (4), the rule of mixtures, was introduced to quantitatively analyze relationships between the elastic modulus, component content, and predict elastic mechanical properties of materials at certain fiber contents.

(4) $$E=E_{f}\cdot V_{f}+E_{p}\cdot (1-V_{f})$$

where *E* is the flexural modulus of bamboo slivers of different layers with fiber volume fraction *V_f_*; and *E_f_* and *E_p_* are the flexural modulus of fibers and parenchyma cells, respectively. Considering that the bamboo sliver with a specific size is a beam structure, the expression of the bending stiffness as a function changing with fiber volume fraction derived from Equations (4) is as follows:

(5) $$\begin{array}{l} S=\frac{EI}{b}=\frac{\left(E_{f}-E_{p}\right)\cdot h^{3}}{12}\cdot V_{f}+\frac{E_{p}\cdot h^{3}}{12}\\ F=S^{-1} \end{array}$$

where $\frac{E_{p}\cdot h^{3}}{12}$ is the bending stiffness of parenchyma cells with thickness of *h*; and $\frac{\left(E_{f}-E_{p}\right)\cdot h^{3}}{12}$ is the difference of bending stiffness between of fibers and parenchyma cells; the thickness (*h*) was kept at 1 mm throughout the study.

**Figure 9:**
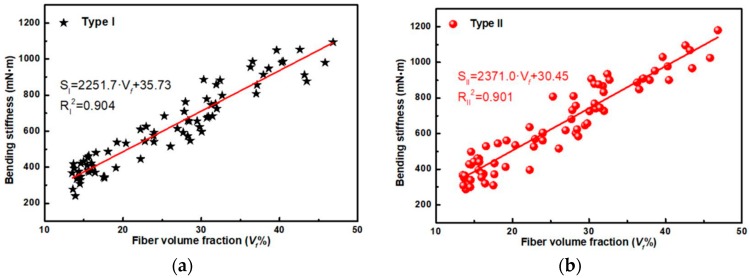
Relationship between bending stiffness and fiber volume fraction under Type I (**a**) and II (**b**).

By Fitting Equation (5) to the test values of the bending stiffness derived for all bamboo slivers samples under different loading Types, the relationship between bending stiffness and fiber volume fraction exhibit a high linear positive correlation (Figure 9).The slope $\frac{\left(E_{f}-E_{p}\right)\cdot h^{3}}{12}$ and intercept $\frac{E_{p}\cdot h^{3}}{12}$ of the line *S*-*V_f_* were obtained, and the theoretical parameters in the model (i.e., *S_p_*, *E_p_*, *S_f_* and *E_f_*) and flexibility of fibers and parenchyma cells with thickness of *h* were calculated (Table 2). 

The flexural modulus and bending stiffness of the fibers ranged between 27–29 GPa and 2200–2400 mN·m, respectively, which was significantly larger than that of parenchyma cells, and the flexibility of the fibers was significantly smaller than that of parenchyma cells. Compared with Type II, the flexibility of the fiber under Type I (0.44 × 10^−3^ mN^−1^·m^−1^) was slightly higher, while the flexibility of the parenchyma cells (27.99 × 10^−3^ mN^−1^·m^−1^) slightly lower. This indicated indicates that bamboo has higher flexibility under natural loading conditions. This is of significance for the preparation and optimization of the WPCs because more rigid pipes can wound from bamboo slivers under inside tension (Type II) instead of non-uniform winding that chaotically and disorderly puts bamboo slivers under both Type I and Type II loading. The theoretical flexural modulus of parenchyma cells (*E_p_*) was consistent with bending test value (0.37 ± 0.11 GPa) of An [9] which were obtained by manual peeling, but significantly lower than tensile modulus (3.7 ± 0.4 GPa) that was obtained by Amada [20], which was related to the anisotropy, bamboo species and test methods, etc. The theoretical *E_f_* was higher than the indentation modulus (22.8 ± 2.8 GPa) measured by nano-indentation and was similar to tensile modulus (30.1 ± 3.0 GPa) by micro-tensile testing [21], in which it related to various factors such as growth environment of bamboo, test conditions, and error. Therefore, in view of the high quality of the fit (R_I_^2^ = 0.904, R_II_^2^ = 0.901 in Figure 9) between the bending stiffness and the fiber volume fraction and close compatibility between the theoretical value of the flexural modulus with fibers and parenchyma cells and experimental values of that measured by others, the validity of the introduced rule of mixtures for bending stiffness could be verified. At the same time, the model could also provide theoretical guidance for the study of mechanical properties of natural FG materials [22] (including bamboo, bones and shells, etc.) as well as bionics and preparation, where WPCs could finally achieve excellent flexible bending behavior without breaking under the extreme external loading.

## 4. Conclusions

Bamboo material’s remarkable graded flexibility is stemmed from the concurrent graded distribution and gradient variation of cell size of tougher fibers embedded in weaker parenchyma cells along the thickness of bamboo culm. 

As the fiber volume fraction increases from the inside to the outside, the flexibility of the bamboo slivers gradually decreases. Moreover, gradient variation of the size of fibers and parenchyma cells also contributes to this trend of flexibility. 

Due to the variation of the shape of the vascular bundles, the bamboo slivers demonstrated an obvious difference in directional flexibility under different loading Types. Relatively small difference of flexibility in the inner layer is attributed to symmetrical structure of the vascular bundles, while the difference in the outer layer is larger due to the arrow structure of the vascular bundles pointed from the outside to the inside.

A numerical model for bending stiffness with high fitness and close compatibility has been adopted to validate its relationship to the fiber volume fraction, which helps to understand the mechanical design and predict mechanical properties of FG materials for bionics purpose.

This combination of soft inner layers and rigid outer layers due to the change in concentration of vascular bundles can be used to improve how BWC pipes are manufactured by improving how the bamboo sliver is layered up in the pipe. So by no longer winding BWC pipes with disregard to orientation of the vascular bundles where a non-uniform random structure would exist an engineered lay-up can be used to optimize the vascular bundles orientation to help better improved the flexural and stiffness properties of the pipe. By constructing the BWC pipes in this manner the process of manufacturing these pipes can be improved and the variation in the pipe properties can be reduced allowing for improved quality control of their production.

## Figures and Tables

**Table 1:** The physical and mechanical properties of bamboo slivers under different bending Types.

Sample	Air-dried Density (ρ) (g/cm^3^)	Fiber Volume Fraction (*V_f_*) (%)	Loading Modes	Bending Stiffness (S) (mN·m)	Bending Flexibility (F) (mN^−1^·m^−1^ × 10^−3^)	Difference of Flexibility (%)
B1	0.55 ± 0.03 ^a^	14.9 ± 2.7 ^a^	Type I	349 ± 48 ^a^	2.92 ± 0.38 ^a^	3.05
			Type II	359 ± 46 ^a^	2.83 ± 0.48 ^a^	
B2	0.60 ± 0.03 ^b^	15.8 ± 2.8 ^a^	Type I	434 ± 61 ^b^	2.35 ± 0.34 ^b^	4.89
			Type II	450 ± 65 ^b^	2.23 ± 0.31 ^b^	
B3	0.67 ± 0.03 ^c^	26.1 ± 2.7 ^b^	Type I	586 ± 39 ^c^	1.71 ± 0.11 ^c^	4.57
			Type II	619 ± 69 ^c^	1.63 ± 0.18 ^c^	
B4	0.72 ± 0.03 ^d^	30.3 ± 2.1 ^c^	Type I	737 ± 71 ^d^	1.37 ± 0.12 ^d^	6.19
			Type II	790 ± 91 ^d^	1.28 ± 0.16 ^d^	
B5	0.80 ± 0.03 ^e^	38.9 ± 5.8 ^d^	Type I	942 ± 71 ^e^	1.07 ± 0.08 ^e^	5.31
			Type II	996 ± 86 ^e^	1.01 ± 0.08 ^e^	

^a, b, c, d, e^ Different letters in the same column indicate significant differences among layers. (*p* < 0.05).

**Table 2:** Predicting value of mechanical properties of fibers and parenchyma cells by a rule of mixture.

Types	Bending Stiffness (*S*) (mN·m)		Flexural Modulus (*E*) (GPa)		Flexibility (*F*) (mN^−1^·m^−1^ × 10^−3^)	
	Parenchyma Cells	Fibers	Parenchyma Cells	Fibers	Parenchyma Cells	Fibers
Type I	35.73	2287	0.429	27.45	27.99	0.44
Type II	30.45	2401	0.365	28.82	32.84	0.42
